# Assessment of Breathomics Testing Using High-Pressure Photon Ionization Time-of-Flight Mass Spectrometry to Detect Esophageal Cancer

**DOI:** 10.1001/jamanetworkopen.2021.27042

**Published:** 2021-10-05

**Authors:** Qi Huang, Shaodong Wang, Qingyun Li, Peiyu Wang, Jianfeng Li, Shushi Meng, Hang Li, Hao Wu, Yu Qi, Xiangnan Li, Yang Yang, Song Zhao, Mantang Qiu

**Affiliations:** 1 Department of Thoracic Surgery, The First Affiliated Hospital of Zhengzhou University, Zhengzhou, China; 2 Department of Thoracic Surgery, Peking University People’s Hospital, Beijing, China; 3 Breax Laboratory, PCAB Research Center of Breath and Metabolism, Beijing, China; 4 Department of Thoracic Surgery, Shenzhen Second People’s Hospital, The First Affiliated Hospital of Shenzhen University, Shenzhen, China

## Abstract

**Importance:**

A triage test is needed to increase the detection rate for esophageal cancer.

**Objective:**

To investigate whether breathomics can detect esophageal cancer among patients without a previous diagnosis of cancer using high-pressure photon ionization time-of-flight mass spectrometry (HPPI-TOFMS).

**Design, Setting, and Participants:**

This diagnostic study included participants who planned to receive an upper endoscopy or surgery of the esophagus at a single center in China. Exhaled breath was collected with a self-designed collector and air bags before participants underwent these procedures. Sample collection and analyses were performed by trained researchers following a standardized protocol. Participants were randomly divided into a discovery data set and a validation data set. Data were collected from December 2020 to March 2021.

**Exposures:**

Breath samples were analyzed by HPPI-TOFMS, and the support vector machine algorithm was used to construct a detection model.

**Main Outcomes and Measures:**

The accuracy of breathomics was measured by the sensitivity, specificity, accuracy, positive predictive value, negative predictive value, and area under the receiver operating characteristic curve.

**Results:**

Exhaled breath samples were obtained from 675 patients (216 [32%] with esophageal cancer; 459 [68%] with noncancer diseases). Of all patients, 206 (31%) were women, and the mean (SD) age was 64.0 (11.9) years. In the validation data set, esophageal cancer was detected with an accuracy of 93.33%, sensitivity of 97.83%, specificity of 83.72%, positive predictive value of 94.74%, negative predictive value of 92.78%, and area under the receiver operating characteristic curve of 0.89. Notably, for 16 patients with high-grade intraepithelial neoplasia, 12 (75%) were predicted to have esophageal cancer.

**Conclusions and Relevance:**

In this diagnostic study, testing breathomics using HPPI-TOFMS was feasible for esophageal cancer detection and totally noninvasive, which could help to improve the diagnosis of esophageal cancer.

## Key Points

**Question:**

Could esophageal cancer be detected by testing breathomics using high-pressure photon ionization time-of-flight mass spectrometry (HPPI-TOFMS)?

**Findings:**

In this diagnostic study of 675 patients with and without cancer, esophageal cancer was accurately detected by testing breathomics with a sensitivity of 97.83% and specificity of 83.72% in the validation data set.

**Meaning:**

These findings suggest that testing breathomics could help to improve the diagnosis of esophageal cancer.

## Teaser

This diagnostic study investigates whether breathomics can detect esophageal cancer among patients without a previous diagnosis of cancer using high-pressure photon ionization time-of-flight mass spectrometry.

## Introduction

Esophageal cancer is the sixth leading cause of cancer-related death worldwide.^1^ It is estimated that nearly half of new esophageal cancer cases are in China.^1^ Approximately 80% of patients with esophageal cancer are diagnosed at a late stage, resulting in poor 5-year survival.^2^ Upper endoscopy is the primary option for esophageal cancer detection and screening; however, endoscopy is not suitable for population-based screening because it is invasive, expensive, and not sensitive among patients without symptoms.^3^ Esophageal cancer may be missed at endoscopy in as many as 7.8% of patients who are subsequently diagnosed with cancer.^4^ Thus, a sensitive, noninvasive, and inexpensive tool is urgently needed to detect esophageal cancer at an early stage.

Dysregulated cellular metabolism is a hallmark of cancer,^5^ and various altered metabolic pathways have been identified in esophageal cancer.^6^ Breathomics, focusing on metabolites in exhaled breath, is a branch of metabolomics and offers the possibility of noninvasive disease diagnoses and therapeutic monitoring.^7^ Several studies have demonstrated that Barrett esophagus can be detected by breathomics testing with an electronic nose,^8,9^ but very few studies have been conducted to analyze breathomics in esophageal cancer.^3^ Recently, several kinds of online mass spectrometry have been used to analyze exhaled breath, such as selected-ion-ﬂow-tube mass spectrometry (SIFT-MS),^10^ proton-transfer reaction MS (PTR-MS),^11,12^ and secondary electrospray ionization MS (SESI-MS).^13^ To simplify breathomics testing procedures and increase the robustness of breathomics results, we substantially optimized direct online mass spectrometry and high-pressure photon ionization time-of-flight mass spectrometry (HPPI-TOFMS) and established standardized breath sampling procedures.^14^

In this study, we postulate that patients with esophageal cancer might have distinct breathomics and that testing breathomics using HPPI-TOFMS could offer a noninvasive, feasible, and accurate triage test for esophageal cancer detection.

## Methods

### Patient Recruitment

This study was reported following the Standards for Reporting of Diagnostic Accuracy (STARD) reporting guideline.^15^ A prospective specimen collection, retrospective masked evaluation design^16^ was used. This study was approved by the ethics committee of the First Affiliated Hospital of Zhengzhou University and has been registered in the Chinese Clinical Trial Registry (ChiCTR2000040966). Written informed consent was obtained from all participants.

All participants were recruited at the First Affiliated Hospital of Zhengzhou University. Participants were recruited according to the following inclusion criteria: (1) older than 18 years, (2) planning to receive upper endoscopy or surgery of the esophagus, and (3) no history of cancer within 5 years and no previous anticancer treatment. Participants were excluded according to the following criteria: (1) patients with at least 2 of the following were considered to have active infection and were excluded: symptoms with fever, cough or sputum; procalcitonin level greater than 0.05 ng/mL, white blood cell count greater than 9500/μL or less than 3500/μL (to convert to ×10^9^ per liter, multiply by 0.001), C-reactive protein level of 0.5 mg/dL or greater (to convert to milligrams per liter, multiply by 10.0), or absolute neutrophil count greater than 6300/μL or less than 1800/μL (to convert to ×10^9^ per liter, multiply by 0.001); (2) patients with chronic liver disease complicating abnormal biochemical markers (albumin, prothrombin time, or bilirubin) were excluded; and (3) patients with chronic kidney disease complicating serum creatinine levels of greater than 1.3 mg/dL (to convert to micromoles per liter, multiply by 88.4). For all participants, clinical data, pathological diagnoses, and demographic data were collected from medical records and questionnaires.

### Exhaled Breath Collection

All exhaled breath samples were collected by trained investigators following the same protocol. All participants fasted for at least 6 hours before breath collection. To reduce potential confounding factors, all participants were asked to not ingest spicy food, alcohol, or coffee the night before exhaled breath collection.

Exhaled breath was collected in Tedlar air bags (DuPont de Nemours). The night before breath collection, the Tedlar bags were baked at 60 °C for 3 hours to fully release possible contaminants and continuously purged with high-purity nitrogen 4 times. Participants first gargled with pure water and then performed a single deep nasal inhalation followed by complete exhalation via their mouth into Tedlar bags. A total of 1000 mL of exhaled breath was collected from each participant. Breath samples were collected in a fixed room, and environment air was also collected before and after sample collection of participants. Ambient background air data were subtracted from that of exhaled breath samples, and the obtained data were used for further analysis. The self-designed sampling equipment including a CO_2_ sensor was used for breath sampling as previously reported.^14^ The CO_2_ sensor was used to ensure that alveolar air was collected: exhaled breath collection began once the CO_2_ sensor detected a CO_2_ concentration exceeding 4%. All air bags were delivered to the laboratory and detected within 4 hours. Exhaled breath was collected before esophageal surgery or endoscopy. The data analysis team was masked to the clinical diagnoses, and clinicians who performed surgery and endoscopy were also masked to the results of breath testing.

### MS Detection

Breatha (Shenzhen Breatha Biological Technology) HPPI-TOFMS was used. Exhaled breath air was directly introduced into the ionization region through 250 μm (inner diameter, 0.60 m) stainless steel capillary from Tedlar bags. The HPPI-TOFMS consisted of a vacuum ultraviolet lamp-based HPPI ion source and an orthogonal acceleration TOF mass analyzer. The TOF mass analyzer had a mass resolution of 4000 (full width at half maximum) at a mass to charge ratio of 92, which was achieved with a 0.4 m ﬁeld-free drift tube. The TOF signals were recorded by a 400 picosecond time-to-digital converter rate at 25 kHz, and all the mass spectra were accumulated for 60 seconds. The pressure in the HPPI ion source was set at 500 Pa, and 2 capillaries were arranged in the ion source. To eliminate condensation of exhaled volatile organic compounds and minimize possible surface adsorption, the stainless-steel capillary was heated to 100 °C, and the HPPI ion source was heated to 60 °C. Mass spectrum peaks with a mass to charge ratio of greater than 350 were recorded, and 31 666 data points were extracted from each sample.

### Data Analysis

The support vector machine (SVM) algorithm was used for feature selection and model construction. In this work, the SVM is the official LinearSVM package,^24^ eg, Liblinear version 2.2.0.

Each breath sample had 31 666 data points, and we performed feature selection to choose the important features. To select the important features, we constructed a feature weight to measure the importance of each feature. Therefore, we first performed 500 iterations of a 4-fold cross-validation on the discovery data set, in which 540 samples were randomly divided into a training set of 405 samples (130 cancer and 275 noncancer) and a test set of 135 samples (43 cancer and 92 noncancer). During each training, square root normalization and L_2 normalization were applied as the preprocessing strategy to normalize the original data. The feature weight was obtained by averaging the SVM weight among all 500 iterations. The regularization parameter *C* of SVM was set to 1.0 in these analyses. After obtaining the feature weight, we performed feature selection by selecting the most importance features; the top *K* features means that we selected the first K importance features among all 31 666 datapoints. With the top *K* features, we conducted 500 iterations of a 4-fold cross-validation on the discovery data set with SVM, using the same division of samples as previously. The validation performance of the selected top *K* features was obtained by performing the average operation among all 500 iterations. *K* was increased from 1 to 200 in this analysis, which means that we successively verified the role of the first 200 features in cancer recognition. The results showed that the top 138 features obtained the highest performance. With the selected top 138 features, we constructed the final model based on whole discovery data set with *C* set at 1 and performed a final evaluation on the validation data set and high-grade intraepithelial neoplasia (HGIN) samples. Because the SVM model presumably produces a continuous prediction, the cutoff point was set as 0 for applying the trained SVM to predict esophageal cancer. The patient was considered to have esophageal cancer if the score was greater than 0.

### Statistical Analysis

Sensitivity, specificity, accuracy, positive predictive value, and negative predictive value were calculated to evaluate the diagnostic performance of the breath test. Receiver operating characteristic (ROC) curves and precision recall curves were generated, and the area under the ROC curve (AUC) was also calculated to evaluate the classification performance of the breath test. Baseline characteristics were analyzed with independent *t* tests or Fisher exact tests. A 2-sided *P* < .05 was considered statistically significant. All statistical analyses were performed using SPSS statistical software version 24.0 (IBM Corp).

## Results

In summary, exhaled breath samples were obtained from 675 patients (216 [32%] with esophageal cancer; 459 [68%] with noncancer diseases). Of these patients, 206 (31%) were women, and the mean (SD) age was 64.0 (11.9) years. Among patients with esophageal cancer, 203 (94%) were diagnosed with esophageal squamous cell carcinoma, and 13 (6%) were diagnosed with esophageal adenocarcinoma. A total of 137 patients had already undergone esophagoscopy and were diagnosed with esophageal cancer before surgery. There was no significant difference in baseline characteristics between patients with esophageal cancer and the control population with noncancer diseases (Table 1). The percentages of patients using proton pump inhibitors and with comorbidities were not significantly different between the 2 groups. Detailed tumor stages and diagnoses of noncancer diseases are shown in eTable 1 and eTable 2 in the Supplement, respectively.

**Table 1.** Characteristics of 675 Participants

Characteristic	Discovery data set			Validation data set		
	Participants, No. (%)		*P* value	Participants, No. (%)		*P* value
	Benign (n = 367)	Cancer (n = 173)^a^		Benign (n = 92)	Cancer (n = 43)^a^	
Age, mean (SD), y	63.6 (13.0)	65.1 (9.1)	.63	61.8 (13.5)	67.2 (8.2)	.02
Sex						
Men	257 (70.0)	118 (68.2)	.76	65 (70.7)	30 (69.8)	.53
Women	110 (30.0)	55 (31.8)		27 (29.3)	13 (30.2)	
BMI, mean (SD)	23.1 (3.7)	23.8 (3.0)	.008	23.1 (4.9)	23.4 (2.3)	.98
TNM stage						
IA, IB	NA	69 (39.9)	NA	NA	12 (27.9)	NA
IIA, IIB	NA	62 (35.8)		NA	22 (51.2)	
IIIA, IIIB, IIIC	NA	33 (19.1)		NA	6 (14.0)	
IVA	NA	9 (5.2)		NA	3 (7.0)	
Smoking						
Current	42 (11.4)	26 (15.0)	.24	13 (14.1)	6 (14.0)	.71
Former	55 (15.0)	32 (18.5)		16 (17.4)	5 (11.6)	
Never	270 (73.6)	115 (66.5)		63 (68.5)	32 (74.4)	
Alcohol use						
Current	90 (24.5)	52 (30.1)	.04	22 (23.9)	16 (37.2)	.29
Former	36 (9.8)	26 (15.0)		15 (16.3)	5 (11.6)	
Never	241 (65.7)	95 (54.9)		55 (59.8)	22 (51.2)	
Bad eating habits^b^	17 (4.6)	17 (9.8)	.04	5 (5.4)	0 (0.0)	.18
Pulmonary disease	26 (7.1)	16 (9.2)	.39	8 (8.7)	2 (4.7)	.50
Diabetes	41 (11.2)	27 (15.6)	.17	13 (14.1)	5 (11.6)	.79
Cardiovascular disease	66 (18.0)	47 (27.2)	.02	20 (21.7)	10 (23.3)	.50
Liver disease	13 (3.5)	10 (5.8)	.26	1 (1.1)	0 (0.0)	.68
Kidney disease	4 (1.1)	4 (2.3)	.45	3 (3.3)	1 (2.3)	.62
PPI use	51 (13.9)	18 (10.4)	.27	10 (10.9)	8 (18.6)	.28
Statin use	26 (7.1)	17 (9.8)	.31	4 (4.3)	2 (4.7)	.62
Antihypertension medications	51 (13.9)	43 (24.9)	.002	24 (26.1)	7 (16.3)	.27
Diabetes medications	34 (9.3)	20 (11.6)	.44	12 (13.0)	7 (16.3)	.79
Aspirin	29 (7.9)	18 (10.4)	.41	4 (4.3)	7 (16.3)	.04

Abbreviations: BMI, body mass index (calculated as weight in kilograms divided by height in meters squared); NA, not applicable; PPI, proton pump inhibitor.

^a^ Esophageal cancer and high-grade intraepithelial neoplasia were combined.

^b^ Bad eating habits defined as a preference for high-temperature food or pickled foods or eating too quickly.

Only 3 invited patients declined to participate in the test, resulting in a patient acceptability rate of 99.6% to complete the study. No adverse events were observed. We excluded 82 patients who did not meet the inclusion criteria: 33 (40%) had symptoms of active infection (eg, fever, cough, sputum) and received antibiotic therapy, 31 (38%) had liver or kidney dysfunction indicated by biochemical tests, 9 (11%) had previous cancer, 5 (6%) were receiving anti–*Helicobacter pylori* therapy, and 4 (5%) had bronchiectasis.

**Figure 1.**
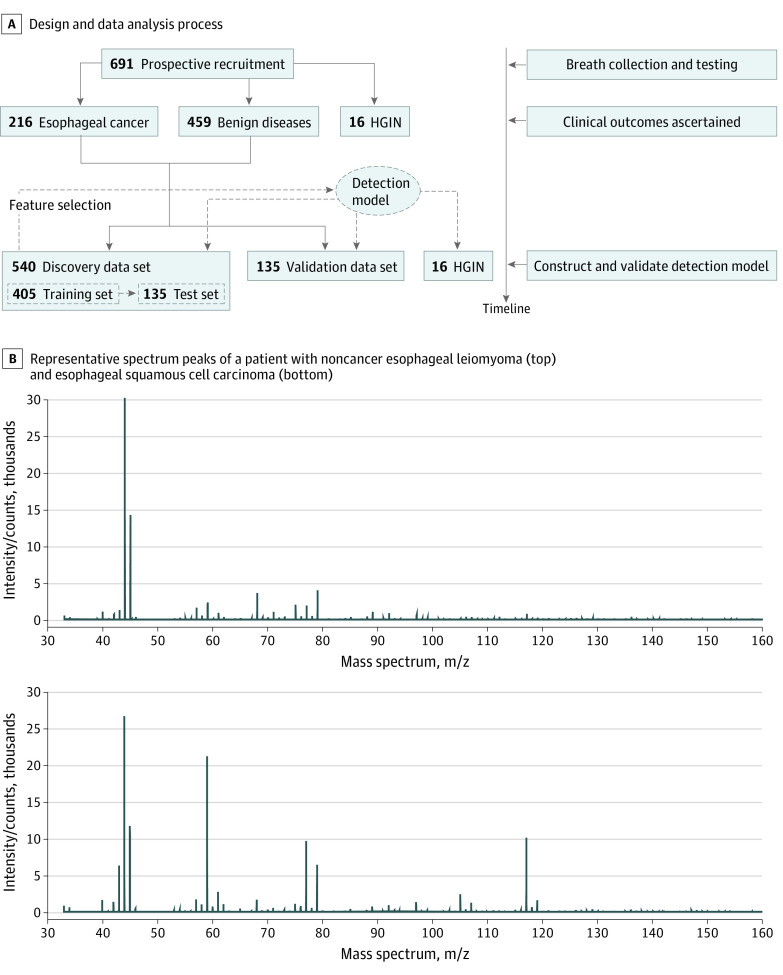
Flowchart of Study Design and Representative Spectrum Peaks of Subgroups HGIN indicates high-grade intraepithelial neoplasia; m/z, mass to charge ratio.

The study design and data analysis process are shown in Figure 1A. We randomly split esophageal cancer and noncancer samples into a discovery data set (540 participants) and a validation dataset (135 participants). The discovery data set, which contained 367 participants with noncancer diseases and 173 participants with esophageal cancer, was used for feature selection and model construction. The validation dataset contained 92 noncancer samples and 43 cancer samples and was used for model validation. HGIN is a precancerous state worthy of independent analysis; therefore, HGIN samples were not used for feature selection. However, the predictive accuracy was evaluated using HGIN samples.

**Figure 2.**
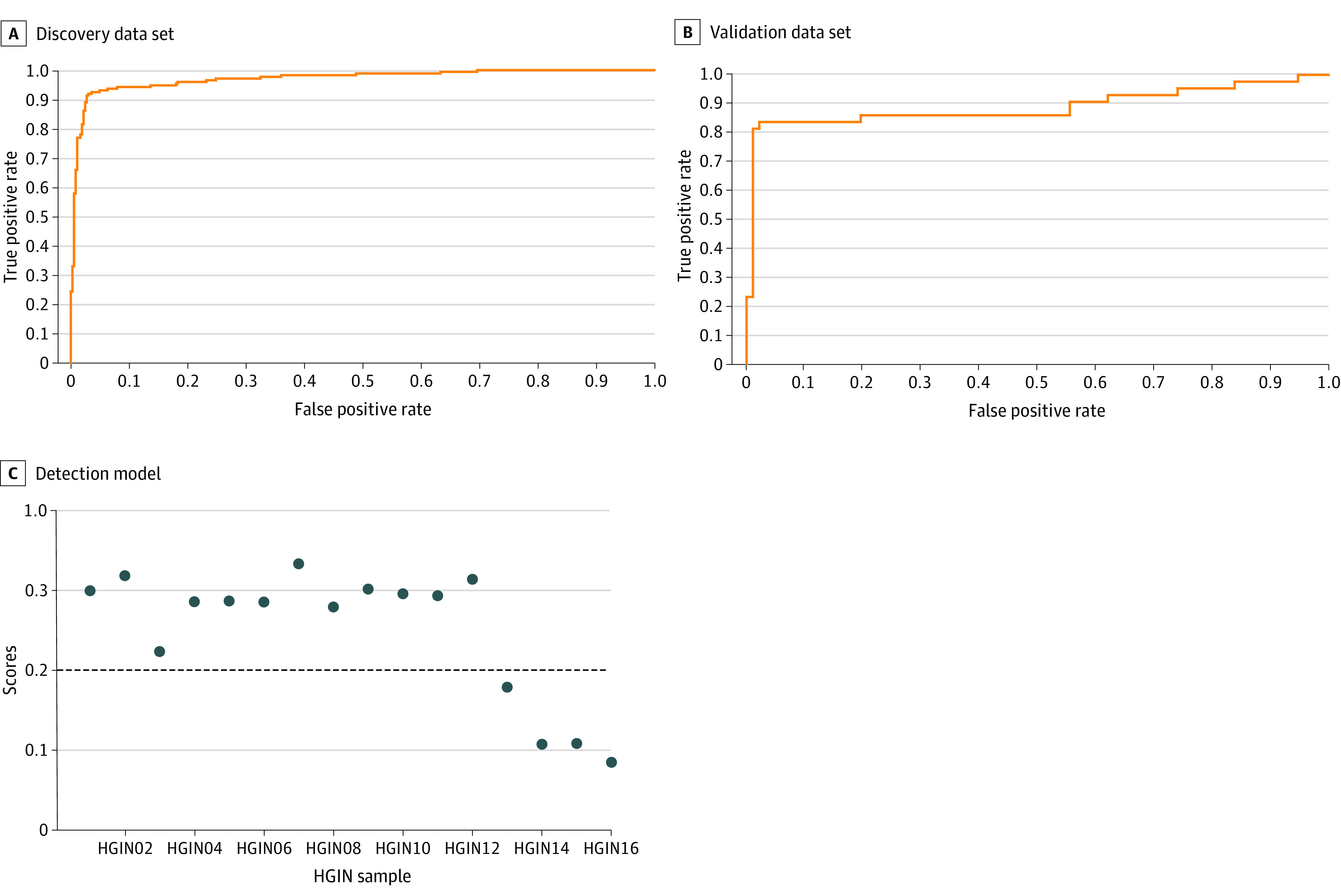
Area Under the Receiver Operating Characteristic Curve for Esophageal Cancer and the Detection Rate for High-Grade Intraepithelial Neoplasia (HGIN) C, Twelve patients with HGIN were tested as having esophageal cancer. Samples with scores greater than 0 were classified as cancerous.

Spectrum peaks of esophageal cancer and noncancer diseases have distinct patterns (Figure 1B). Using the SVM algorithm, we selected 138 features from 31 666 data points to construct and validate the detection model (eTable3 in the Supplement). Esophageal cancer was successfully detected in the discovery data set with a sensitivity of 97.55%, specificity of 86.13%, accuracy of 93.89%, positive predictive value of 94.30%, negative predictive value of 93.72%, and AUC of 0.97 (Figure 2A). Then, in the validation data set, the detection model achieved a sensitivity of 97.83%, specificity of 83.72%, accuracy of 93.33%, PPV of 94.74%, NPV of 92.78%, and AUC of 0.89 (Figure 2B). By multivariable logistic regression analyses, we found that the score of the detection model was an independent predictor of esophageal cancer adjusted for age, sex, and other factors (odds ratio, 81.85; 95% CI, 39.71-168.73; *P* < .001) (Table 2).

**Table 2.** Multivariable Logistic Regression Analyses for 675 Participants

Characteristic	OR (95% CI)	*P* value
Sex	1.19 (0.54-2.61)	.67
Age	1.02 (0.98-1.06)	.35
BMI	1.02 (0.92-1.13)	.70
Score of detection model^a^	81.85 (39.71-168.73)	<.001
Smoking	1.11 (0.68-1.79)	.70
Alcohol use	0.87 (0.55-1.37)	.54
Bad eating habits^b^	1.02 (0.21-5.09)	.98
Pulmonary disease	0.98 (0.20-4.82)	.98
Diabetes	0.54 (0.18-1.58)	.26
Cardiovascular disease	0.53 (0.23-1.24)	.14
Liver disease	0.38 (0.05-2.62)	.32
Kidney disease	1.72 (0.10-28.44)	.71
PPI use	1.02 (0.34-3.04)	.97
Statin use	0.48 (0.14-1.65)	.25
Antihypertensive medications	0.38 (0.15-0.92)	.03
Diabetes medications	0.70 (0.23-2.16)	.54
Aspirin	0.18 (0.06-0.60)	.005

Abbreviations: BMI, body mass index; OR, odds ratio; PPI, proton pump inhibitor.

^a^ Esophageal cancer status as the dependent variable. Patients with high-grade intraepithelial neoplasia were not included in regression analyses.

^b^ Bad eating habits defined as a preference for high-temperature food or pickled foods or eating too quickly.

HGIN samples were used for validation. HGIN was also detected by breathomics, as 12 of 16 patients with HGIN (75%) were classified as having esophageal cancer (Figure 2C).

## Discussion

Breathomics is a branch of metabolomics. Human breathomics involves thousands of low molecular weight metabolites, also called volatile organic compounds, and has been used for the early detection of cancer.^10,17^ Cancer-derived metabolites can circulate to the lungs and diffuse into the alveoli via gaseous exchanges between blood and air.^18^ Similar to the rapidly developing liquid biopsy techniques in cancer,^19,20^ such as circulating cell-free DNA, circulating tumor cells, circulating micro-RNAs, or even exosomes in circulation,^21^ metabolites in exhaled breath also contain valuable information about what happens within our bodies. Thus, testing breathomics holds great potential as an emerging breath biopsy.

Gas-chromatography MS and electronic noses are 2 standard techniques to analyze exhaled breath,^17^ but they have certain drawbacks that limit their clinical application. Much progress has been made in testing breathomics, and many new analytical methods have been used, such as SIFT-MS, PTR-MS, and SESI-MS.^3,11,12,13^ HPPI-TOFMS is a direct online MS that does not require pretreatment of exhaled breath, holds great tolerance for humidity, detects rapidly (60 seconds per sample), and limits the detection down to 52 × 10^-9^ g/m^3^ for benzene.^22,23^ Thus, HPPI-TOFMS appears to be a useful tool to explore human breathomics.

Compared with previous studies with Barrett esophagus^8,9^ and esophagogastric cancer,^3^ we included more eligible participants and achieved better diagnostic accuracy. Markar et al^3^ included only 53 patients with esophageal cancer in their study, while 232 patients with esophageal cancer were included in the current study, which increases the statistical power of our detection model. Additionally, Peters et al^9^ reported a patient acceptability rate of 91.4% in their study; however, the patient acceptability rate was 99.6% in our study. One possible explanation is that sampling electronic noses requires approximately 5 minutes for an individual, but it takes approximately 10 seconds with our sampling equipment.

Markar et al^3^ performed a decision workshop and located breath testing in primary care to triage patients with nonspecific symptoms to undergo endoscopy. It is highly possible that testing breathomics as a triage test performed before endoscopy could substantially increase the detection rate of esophageal cancer and reduce the number of negative endoscopies. An ideal triage test requires high sensitivity and positive predictive value. In the validation dataset, our breath test achieved 97.83% sensitivity, 83.72% specificity, a positive predictive value of 94.74%, and a negative predictive value of 92.78%, which meet the requirements of a good triage test. Furthermore, we developed a easy and user-friendly breath sampling and detection procedure, and a person without a medical background could perform the breath test with simple training. The noninvasiveness and feasibility of breathomics suggest that testing breathomics would be a potential option for esophageal cancer detection and screening.

### Limitations

This study has limitations. One potential limitation is that we could not characterize exact molecules of the 138 selected features because it requires complex analytical chemistry to identify the structure of a single metabolite, and this was beyond the aim of the current study. Further identification of these metabolites at the molecular level and characterization of their metabolic pathways in esophageal cancer are required to increase the robustness and repeatability of our model on other platforms. Notably, the breath test also showed good predictive performance for the precancerous state, HGIN. However, this finding should be interpreted with caution given that no patients with HGIN were included for model construction, and the sample size was small. Additionally, comorbidities and medication information were retrieved from questionnaires, which may be affected by recall bias.

## Conclusions

In this diagnostic study, we demonstrated as a proof of concept that testing breathomics is feasible and highly acceptable among patients receiving upper endoscopy. Esophageal cancer has distinct breathomics, and a simple breath test with the HPPI-TOFMS platform accurately detected esophageal cancer among patients with noncancer diseases in the validation data set. Given the advantages of high acceptability, tolerability, noninvasiveness, and high accuracy, testing breathomics with HPPI-TOFMS may be a promising noninvasive approach for esophageal cancer detection and offers the possibility for breath biopsy.

## Notes

Supplement. **eTable 1.** Pathological Characteristics of 216 Patients With Esophageal Cancer **eTable 2.** Diagnoses of 459 Participants With Noncancer Diseases **eTable 3.** Mass Spectrometry Results of the 138 Selected Features
