# Supplementary material for: Assessment of Breathomics Testing Using High-Pressure Photon Ionization Time-of-Flight Mass Spectrometry to Detect Esophageal Cancer
Source: JAMA Netw Open. 2021 Oct 5;4(10):e2127042. doi: 10.1001/jamanetworkopen.2021.27042 (PMC8493434; doi:10.1001/jamanetworkopen.2021.27042)
Supplement: Supplement. — eTable 1. Pathological Characteristics of 216 Patients With Esophageal Cancer eTable 2. Diagnoses of 459 Participants With Noncancer Diseases eTable 3. Mass Spectrometry Results of the 138 Selected Features [file jamanetwopen-e2127042-s001.pdf]

Supplemental Online Content

Huang Q, Wang S, Li Q, et al. Assessment of breathomics testing using high-pressure photon ionization time-of-flight mass spectrometry to detect esophageal cancer. *JAMA Netw Open.* 2021;4(10):e2127042. doi:10.1001/jamanetworkopen.2021.27042

eTable 1. Pathological Characteristics of 216 Patients With Esophageal Cancer

eTable 2. Diagnoses of 459 Participants With Noncancer Diseases

eTable 3. Mass Spectrometry Results of the 138 Selected Features

This supplemental material has been provided by the authors to give readers additional information about their work.

© 2021 Huang Q et al. *JAMA Network Open.*

eTable1. Pathological Characteristics of 216 Patients With Esophageal Cancer

Tumor-Related Factor	Patients, n (%)
Histological Subtypes	
ESCC	203(94.0)
EAC	13(6.0)
TNM Stage	
IA, IB	81(37.5)
IIA, IIB	84(38.9)
IIIA, IIIB, IIIC	39(18.1)
IVA	12(5.6)
Tumor Location	
Upper	29(13.4)
Middle	101(46.8)
Lower	86(39.8)
Primary Tumor(T)	
T1	61(28.3)
T2	82(38.0)
T3	62(28.7)
T4	11(5.1)
Regional Lymph Nodes(N)	
N0	120(55.6)
N1	62(28.7)
N2	29(13.4)
N3	5(2.3)
Pathological Grade(G)	
G1	36(16.7)
G2	112(51.9)
G3	68(31.5)

ESCC: esophageal squamous cell carcinoma; EAC: esophageal adenocarcinoma

© 2021 Huang Q et al. *JAMA Network Open.*

eTable 2. Diagnoses of 459 Participants With Noncancer Diseases

Diagnoses	Patients, n (%)
Esophagitis	210(45.8)
Chronic Esophagitis	173(37.7)
Reflux Esophagitis	37(8.1)
Gastritis	157(34.2)
Chronic Gastritis	116(25.3)
Erosive Gastritis	41(8.9)
Protrusive Oesophageal Lesion	28(6.1)
Leiomyoma	17(3.7)
Papilloma	6(1.3)
Cyst	3(0.7)
Polyp	2(0.4)
Gastric Polyp	44(9.6)
Gastroduodenal Ulcer	20(4.4)

© 2021 Huang Q et al. *JAMA Network Open.*

eTable 3. Mass Spectrometry Results of the 138 Selected Features

Top-K	m/z
1	55.029495
2	30.0967349
3	68.0525396
4	30.051368
5	28.0203118
6	30.1032187
7	55.0382659
8	37.0306355
9	88.0039019
10	28.0140568
11	30.0448898
12	76.0902141
13	37.0234429
14	88.0149957
15	76.0798997
16	43.983383
17	127.138622
18	43.9912235
19	68.0622942
20	28.0265675
21	76.1005292
22	154.193287
23	79.0793661
24	68.0330323
25	117.077944
26	55.0470376
27	85.0996045
28	117.026764
29	98.0956272
30	28.070377
31	88.0260902
32	78.9952639
33	154.178601
34	76.069586

© 2021 Huang Q et al. *JAMA Network Open.*

35	93.1010368
36	127.151957
37	154.119864
38	19.0307733
39	87.9928087
40	39.0277517
41	57.0652434
42	107.02797
43	84.0989584
44	93.0896267
45	98.0839149
46	106.039261
47	59.0561998
48	174.110599
49	107.040205
50	55.0207247
51	28.0766384
52	85.1105136
53	174.126206
54	37.0378287
55	105.091509
56	58.0249234
57	92.0315478
58	127.125287
59	30.1097032
60	93.1124476
61	58.006912
62	108.045854
63	154.105182
64	107.052441
65	117.103538
66	79.026797
67	79.0478225
68	56.0604463
69	85.088696
70	63.0373213
71	113.094352
72	127.165293
73	109.996832

© 2021 Huang Q et al. *JAMA Network Open.*

74	106.027083
75	57.0563122
76	57.0741753
77	58.0339302
78	76.059273
79	37.0810029
80	19.0359263
81	39.0203675
82	68.0135278
83	117.090741
84	117.141935
85	84.1098032
86	92.0202036
87	31.9862973
88	43.0476115
89	117.013971
90	154.134547
91	30.0578469
92	99.058419
93	140.150767
94	28.0641165
95	103.076662
96	279.253875
97	18.0192411
98	107.015736
99	96.1029886
100	68.0427855
101	41.0319502
102	155.193567
103	99.0701894
104	106.051439
105	154.207973
106	113.132085
107	55.161133
108	131.116486
109	105.079386
110	170.215465
111	139.14449
112	279.273641

© 2021 Huang Q et al. *JAMA Network Open.*

113	55.1523522
114	279.234109
115	19.0462345
116	174.141813
117	112.07793
118	108.058147
119	138.11404
120	90.0569462
121	92.0428929
122	117.039558
123	131.130029
124	139.130539
125	131.102944
126	107.064677
127	57.0205944
128	43.1329712
129	174.094993
130	56.0515942
131	155.134638
132	18.0242549
133	108.033561
134	67.0418722
135	210.206151
136	112.128016
137	98.0722032
138	93.0325868

© 2021 Huang Q et al. *JAMA Network Open.*
